# Identifying Profiles of Anxiety in Late Childhood and Exploring Their Relationship with School-Based Distress

**DOI:** 10.3390/ijerph18030948

**Published:** 2021-01-22

**Authors:** Aitana Fernández-Sogorb, Ricardo Sanmartín, María Vicent, Carolina Gonzálvez

**Affiliations:** Department of Developmental Psychology and Didactics, Faculty of Education, University of Alicante, San Vicente del Raspeig, 03690 Alicante, Spain; aitana.fernandez@ua.es (A.F.-S.); ricardo.sanmartin@ua.es (R.S.); carolina.gonzalvez@ua.es (C.G.)

**Keywords:** anxiety, distress, childhood, health, latent profile analysis

## Abstract

Failure in dealing with anxiety-provoking situations and stressors in the school setting may have negative consequences not only on children’s performance, but also on their well-being in the future. This research aimed to examine the relationship of forms of anxiety (anticipatory anxiety, school-based performance anxiety, and generalized anxiety) with sources (teacher interactions, academic stress, peer interactions, and academic self-concept) and manifestations (emotional, behavioral, and physiological) of school-based distress. Specifically, our objectives were to examine the correlations between anxiety and school-based distress and, using a person-centered approach, to verify whether different anxiety profiles differed in their levels of distress. The Visual Analogue Scale for Anxiety-Revised (VAA-R) and the School Situation Survey (SSS) were administered to 756 Spanish students (*M_age_* = 9.6, *SD* = 1.12); 50.3% were girls. Pearson’s correlation coefficients revealed a positive and significant association between each form of anxiety and each source and manifestation of distress. The latent profile analysis identified three anxiety profiles: High Anxiety, High School-based performance Anxiety, and Low Anxiety. The High Anxiety profile scored significantly higher in all sources and manifestations of distress than the Low Anxiety profile. The High Anxiety profile showed significantly higher scores in peer interactions and emotional and behavioral manifestations of distress than the group High School-based performance Anxiety. Suggestions for intervention strategies according to the risk profile are discussed.

## 1. Introduction

Anxiety is a fear that anticipates possible threats [1]. When the predictions are profuse and lack objectivity, this emotion is experienced with excessive intensity and becomes maladaptive [2,3]. In childhood, the educational center constitutes a safe environment in which students learn from a great number of experiences. However, they may perceive some situations as dangerous. According to Bernstein and Garfinkel [4], children may feel anxiety about arriving at school (i.e., anticipatory anxiety) or anxiety about performing inside the school in social situations such as speaking in front of the class (i.e., school-based performance anxiety), in addition to experiencing generalized anxiety symptoms. Anxious students tend to avoid anxiety-provoking events [5], which may result in school refusal behavior (i.e., resistance to attending school [6]) and low academic achievement [7]. Several studies have concluded that students’ performance may also be particularly affected by school-based distress (e.g., [8,9]). Both anxiety and distress “could lead to difficulties in concentration and lack of motivation and interest” [10] (p. 1354). In this sense, “a stressor is any relevant stimulus that puts a demand on an individual. […] the stress response is subjective and dependent upon the individualized appraisal of the demand. The resultant response is delineated into both distress, the negative, undesirable, and harmful response, and eustress, the positive, desirable, and advantageous response. The two responses are considered to be distinct constructs” [11] (p. 2).

School-based distress is understood as an appraisal that either self-requirements or demands imposed on children by other school agents exceed individual coping resources [12,13]. Historically, sources and manifestations of distress for students have been categorized in different ways (see Matheny et al. [14] for a review). The Helms and Gable [15] categorization is noted for having been widely used in studies with child samples (e.g., [16]). Their categorization comprises four sources of distress: teacher interactions, academic stress, peer interactions, and academic self-concept. Teacher interactions are conceptualized as students’ perceptions of the feelings or attitudes their teachers have developed towards them [15]. Thus, children may manifest distress as a result of teacher–child interactions in which the affectivity is negative [17]. According to Helms and Gable [15], students experience academic stress when they feel distress about taking exams, getting poor grades, or about general academic performance. Peer interactions are considered a source of distress when students perceive that their classmates experience negative feelings towards them [15]. In this sense, desirable peer relationships have been found to contribute to students’ well-being (i.e., less distress symptoms) [18]. Finally, academic self-concept is understood as “the self-perception of a student’s ability in an academic domain” [19] (p. 204). The categorization of school-based distress by Helms and Gable [15] also comprises three manifestations of distress: emotional (e.g., frustration), behavioral (e.g., being hurtful), and physiological (e.g., tremors).

The inability to manage anxiety-provoking situations and stressors in the school setting may not only influence students’ level of academic achievement, but also their capacity to cope with adversity in the future as adults. This fact evidences the need for educational psychologists, teachers, and parents to apply interventions to help children overcome both anxiety and school-based distress [20,21]. There is a growing body of scientific literature suggesting that symptoms of anxiety and distress in school-aged children are associated with factors such as gender, age, socioeconomic status or parental occupation (e.g., [22,23]). However, the relationship between both constructs in childhood remains poorly understood. Therefore, a crucial next step is to examine the association between children’s forms of anxiety and school-based distress, since it would add relevant information for the development of specific programs that favor students’ self-regulation [24].

### 1.1. Anxiety and School-Based Distress

The relationship between anxiety and school-based distress has been analyzed in several works. These studies coincide in finding a positive and significant association of school-based distress with anxiety [25,26,27,28,29,30,31,32,33,34,35], test anxiety [10,36,37,38,39,40,41], and school anxiety [42,43]. Regarding the sample’s characteristics, except for two studies with children [28,42], all of them focused on adolescents or university students. In addition, most of these works were carried out in North American, European, and Asian populations.

From the review of previous empirical evidence, there appear several limitations. Firstly, only three studies [37,42,43] examined the sources of distress (i.e., teacher interactions, academic stress, peer interactions, and academic self-concept) and the manifestations of distress (i.e., emotional, behavioral, and physiological) that comprises the Helms and Gable [15] categorization. Secondly, school-based distress proved to be related to anxiety, test anxiety, and school anxiety. However, no study considered the fact that students may experience generalized anxiety and different forms of anxiety in the school setting (e.g., anticipatory anxiety) [4]. Furthermore, there are only a few works assessing anxiety and school-based distress during childhood [28,42] and none of them used a Spanish sample, even though national studies report high rates of prevalence of both child anxiety (11.8%) [44] and distress symptoms (5.8%) [45]. Finally, a positive and significant association has been identified between both constructs, but it is unknown whether different profiles of child anxiety (i.e., subgroups of children with unobservable heterogeneity) differ in their levels of school-based distress.

### 1.2. Profiles of Child Anxiety

It has become habitual to distinguish anxiety profiles in children with pediatric anxiety disorders (e.g., [46,47]). Apart from clinical studies, less is known about the way in which community samples of students are grouped into profiles of child anxiety. In this sense, possible combinations of forms of anxiety have been analyzed in order to establish different subpopulations of children, each profile being characterized by the weight obtained by the forms of anxiety. 

On the one hand, mathematics anxiety, test anxiety, and generalized anxiety have been examined in two studies. The first one was conducted in the United Kingdom [48]. 903 students between 11 and 13 years (*M* = 148.0 months, *SD* = 4.0 months) were grouped into four profiles: High Anxiety (i.e., mathematics anxiety, test anxiety, and generalized anxiety), Low Anxiety, Academic Anxiety (i.e., mathematics anxiety and test anxiety), and General Anxiety (i.e., generalized anxiety). In addition, 817 students between 8 and 9 years (*M* = 109.4 months, *SD* = 3.7 months) were grouped into four profiles with lower specificity: High Anxiety, Moderate Anxiety, Slight Anxiety, and Low Anxiety. The second work was conducted in Italy [49]. In this case, 664 children (*M_age_* = 9.20, *SD* = 1.13) were also grouped into less specific profiles: High Risk of anxiety (i.e., mathematics anxiety, test anxiety, and generalized anxiety), Average Risk, and Low Risk. 

On the other hand, three studies have identified profiles of Spanish children by considering the forms of anxiety suggested by Bernstein and Garfinkel [4]: anticipatory anxiety, school-based performance anxiety, and generalized anxiety. First, 911 children aged 8 to 12 (*M* = 9.61, *SD* = 1.23) were grouped into four profiles: High Anxiety (i.e., anticipatory anxiety, school-based performance anxiety, and generalized anxiety), High Anxiety School-type (i.e., anticipatory anxiety and school-based performance anxiety), Moderate Anxiety, and Low Anxiety [50]. Secondly, 1287 students between 8 and 11 years (*M* = 9.68, *SD* = 1.20) were grouped into four profiles: High Anxiety, Moderate Anxiety, Low Anxiety School-type, and Low Anxiety [51]. Finally, 1161 children aged 8 to 11 (*M* = 9.72, *SD* = 1.14) were grouped into three profiles: High Anxiety, High School-based performance Anxiety, and Low Anxiety [52]. In sum, two in three works identified a specific profile characterized by different weight obtained by the forms of anxiety in the school setting (i.e., anticipatory anxiety and school-based performance anxiety) and three profiles with the same weight obtained by anticipatory anxiety, school-based performance anxiety, and generalized anxiety. However, one in three works identified three profiles, among which a specific group showed different weight obtained only by school-based performance anxiety. Therefore, there is no consensus on the specificity of anxiety profiles in childhood.

### 1.3. The Current Study

In order to overcome the above mentioned limitations, our purpose was to analyze the relationship between forms of anxiety and school-based distress in a Spanish child sample. Specifically, we examined, in the first place, the correlations between both variables, considering three forms of anxiety (i.e., anticipatory anxiety, school-based performance anxiety, and generalized anxiety) highlighted by Bernstein and Garfinkel [4] and the sources of distress (i.e., interactions, academic stress, peer interactions, academic self-concept) and the manifestations of distress (i.e., emotional, behavioral, and physiological) established by Helms and Gable [15]. Given that a positive and significant association has been found of school-based distress with anxiety (e.g., [35]), test anxiety (e.g., [36]), and school anxiety (e.g., [43]), we expected to find positive and significant correlations between all the forms of anxiety and all the sources and manifestations of distress (Hypothesis 1).

In the second place, we assessed whether different groups of children, defined according to their anxiety profile, differed in their levels of school-based distress. Taking as reference previous studies identifying anxiety profiles in Spanish primary education students [50,51,52], we expected to find a model of latent profiles, in which a specific profile of anxiety in the school setting (i.e., anticipatory anxiety and/or school-based performance anxiety) could be one of them (Hypothesis 2). In addition, our last hypothesis was that the group of students with the highest levels of anticipatory anxiety, school-based performance anxiety, and generalized anxiety showed significantly higher scores in the sources and the manifestations of distress (Hypothesis 3).

## 2. Materials and Methods

### 2.1. Participants and Procedure

The sample of this study was recruited using a multi-stage random cluster sampling, with the primary units being the geographical areas: central, north, south, east, and west of the provinces of Murcia and Alicante (Spain). The secondary units were the schools; one or two centers were randomly and proportionally selected in each geographical area. Thus, a total of 15 public and private educational centers were selected. The tertiary units were the classrooms; from each center, one classroom per academic grade was randomly selected from 3rd to 6th grade of primary education in Spain.

The initial sample was made up of 909 students, of which 153 (16.83%) were excluded because: (a) their parents or legal guardians did not give written consent to participate in the research (*n* = 62, 6.82%); (b) they were unable to understand the self-report measures due to a low reading levels (*n* = 51, 5.61%); or because (c) they did not properly complete the questionnaires (*n* = 40, 4.4%). As a result, a final sample of 756 participants between 8 and 11 years old (*M* = 9.6, *SD* = 1.12) was obtained. Table 1 presents the frequency distribution by gender and age; the sample distribution was uniform (χ^2^_(3)_ = 2.9, *p* = 0.41).

The head of study and the director of each school were interviewed in order to present the goal of the research and suggest them to collaborate with it. Then, a letter indicating the purpose of the study was provided to the parents or legal guardians of the students. This letter was signed by those parents or legal guardians who agreed to participate in the study. Thus, only those children who returned the parental consent were selected. The participants responded the VAA-R and the SSS in their group-class, during school hours, and lasting approximately 45 min (five minutes instructions, 10 min the VAA-R, and 30 min the SSS). During test administration, a duly trained researcher was present to inform about the anonymous and voluntary character of the activity, explain the procedure, and solve any doubts to the participants. This study was carried out in accordance with the Declaration of Helsinki. It was also approved by the Ethics Committee of the University of Alicante (UA-2017-09-05).

### 2.2. Measures

#### 2.2.1. Visual Analogue Scale for Anxiety-Revised (VAA-R)

This scale was developed by Bernstein and Garfinkel [4] and validated in a Spanish child sample by Fernández-Sogorb et al. [50]. Its 11 items are organized into three factors referring to the aforementioned forms of anxiety: anticipatory anxiety (AA), including five items (e.g., “Thinking about going to school on Monday”); school-based performance anxiety (SA), including three items (e.g., “Standing up and speaking in front of class”); and generalized anxiety (GA), including three items (e.g., “How I feel most of the time”). Its visual response scale is made of 10 points (steady vs. nervous). Adequate coefficients of internal consistency were obtained in this study: α = 0.84 (AA), 0.76 (SA), and 0.73 (GA). 

#### 2.2.2. School Situation Survey (SSS)

This 34-item questionnaire was developed by Helms and Gable [15]. Its seven factors correspond to the sources and the manifestations of distress that comprises the categorization above-mentioned. Thus, the SSS contains four factors referring to school-based sources of distress: teacher interactions (TI), including six items (e.g., “Some of my teachers call on me when they know I am not prepared just to embarrass me”); academic stress (AS), including three items (e.g., “I am afraid of getting poor grades”); peer interactions (PI), including six items (e.g., “Other students make fun of me”); and academic self-concept (ASC), including four items (e.g., “I feel that I learn things easily”). The SSS also contains three factors that assess manifestations of distress in school: emotional (E) with six items (e.g., “I feel frustrated”), behavioral (B) with six items (e.g., “I yell at my classmates”), and physiological (PH) with three items (e.g., “I feel sick to my stomach”). The Likert-type response scale consists of 5 points (1 = never; 5 = always). High scores in the factors indicate high levels of school-based distress, except for ASC, since a high score in this factor reflects poor academic self-concept. In this study, adequate reliability values were obtained: α = 0.70 (TI), 0.70 (AS), 0.73 (PI), 0.74 (ASC), 0.88 (E), 0.74 (B), 0.78 (PH).

### 2.3. Data Analyses

Pearson’s correlation coefficients between the three factors of the VAA-R and those from of the SSS were calculated. For these correlations, the effect sizes of statistical significance were considered small when values oscillated between 0.10 and 0.29, moderate between 0.30 and 0.49, and large for values ≥0.50 [53].

Latent Profile Analysis (LPA) was conducted in order to distinguish subpopulations of anxious students based on the three forms of anxiety: AA, SA, and GA. The standardized *z* scores [54] obtained in these factors were used. LPA starts with a classification adjustment for all participants, which leads to a profile. Next, the participants are successively reassigned to an ascending number of profiles. Several fit indices were considered to determine the optimal number of profiles that best fitted to data [55]. In the first place, the lowest values of the Bayesian Information Criteria (BIC) and the Akaike Information Criteria (AIC) indicated the greatest explanatory power of the profile solution. In the second place, a greater precision of the classification was reported by entropy values closer to 1. In the third place, statistical significance was considered as the Vuong-Lo-Mendell-Rubin Likelihood-Ratio Test (LRT) and the Bootstrap Likelihood Ratio Test (BLRT) *p*-values smaller than 0.05. On the other hand, no model with any profile including less than 25 participants classified was considered, since the population examined must be represented through all the profiles [56]. Furthermore, the interpretability of each model was analyzed according to the previous literature on the topic [57,58].

A multivariate analysis of variance (MANOVA) was conducted to identify possible differences among the profiles of child anxiety in the mean levels of the four sources of distress (i.e., TI, AS, PI, ASC) and the three manifestations of distress (i.e., E, B, PH). Eta square was used in order to determine the magnitude of effect. Then, post hoc tests (Bonferroni method) were performed to find among which anxiety profiles there were statistically significant differences. The magnitude of these differences was determined by calculating the Cohen’s *d* index. In this sense, the effect size was considered small when *d* levels were between 0.20 and 0.49, moderate between 0.50 and 0.79, and large for levels ≥0.80 [53].

These statistical analyses were performed by using SPSS, version 26.0 (IBM Corporation, Armonk, NY, USA) and MPlus, version 8 (Muthén & Muthén, Los Angeles, CA, USA).

## 3. Results

### 3.1. Correlations between Anxiety and School-Based Distress

Table 2 shows correlations between the questionnaires used in this study. Positive and significant correlations (*p* < 0.001) of a small magnitude were identified between AA, SA, and GA and all the factors of the SSS, except for the emotional manifestation of distress. Thus, a positive and significant association of a moderate magnitude was found between E and AA, SA, and SA.

### 3.2. Latent Profiles of Anxiety

The fit indices for the five estimated models are presented in Table 3. The AIC values decreased for each model that increased one profile. The BIC values also decreased progressively as the number of profiles increased up to 4 and a tendency to stabilize was identified in BIC values from the four-profile solution. However, four-, five-, and six-profile models were rejected since these solutions included one profile with less than 25 cases and their LRT *p*-values were greater than 0.05. Considering these criteria, the three-profile model was the most parsimonious. In addition, the entropy value of this solution was closer to 1 than that corresponding to the two-profile solution, reporting a greater precision in the classification of 76% of participants.

**Figure 1:**
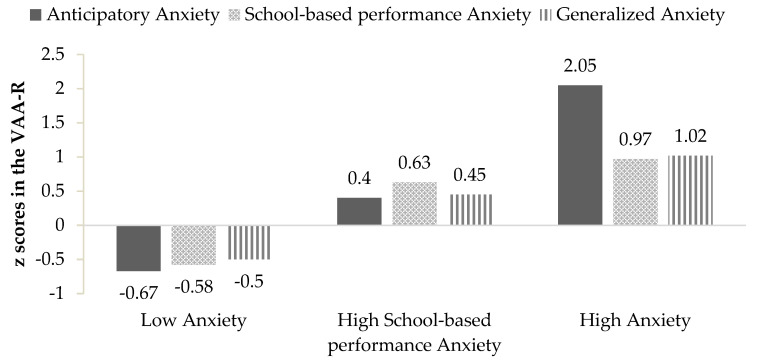
Graphic representation of the three-profile model.

Furthermore, the three-profile model was selected due to a greater interpretability in relation to previous studies. In this sense, the first profile classified 388 participants (51.3%), who reported low levels in AA, SA, and GA. Therefore, it was named Low Anxiety. The second profile included 289 participants (38.2%), who were characterized by high levels of SA and moderate levels of AA and GA, so it was labeled as High School-based performance Anxiety. The third profile, which was called High Anxiety, consisted of 79 participants (10.5%) with high scores in AA, SA, and GA (see Figure 1).

### 3.3. Differences among the Latent Profiles of Anxiety in School-Based Distress

A MANOVA established whether the three profiles of anxiety differed in the mean scores of the seven factors of school-based distress. Statistically significant differences were identified among the latent profiles in all factors of the SSS (Lambda de Wilks = 0.82, *F*_(14,753)_ = 11.30, *p* < 0.001, η^2^ = 0.10). The Low Anxiety profile showed the lowest mean scores in the four sources and the three manifestations of school-based distress, whereas the High Anxiety profile scored the highest in all these factors (see Table 4).

Regarding the post hoc comparisons (see Table 5), the High School-based performance Anxiety profile reported significantly higher scores than the Low Anxiety profile in all sources and manifestations of school-based distress. The magnitudes of these statistical differences were small for all cases, except for the emotional manifestation of distress, whose effect size was of a moderate magnitude. Furthermore, the High Anxiety profile had significantly higher levels than the Low Anxiety profile in all the factors of the SSS. In this case, large and moderate magnitudes were obtained. Finally, the High Anxiety profile scored significantly higher in PI and B than the High School-based performance Anxiety profile, with small magnitude, whereas these statistical differences were identified in E with a moderate magnitude.

## 4. Discussion

The purpose of this study was to examine the relationship between two prevalent constructs in the Spanish children population: anxiety and school-based distress. Firstly, correlations were analyzed. The results revealed a positive and significant association between each form of anxiety (i.e., AA, SA, and GA) and each source (i.e., TI, AS, PI, ASC) and manifestation (i.e., E, B, PH) of distress. This finding corroborates Hypothesis 1 and is in line with previous works that identified positive and significant correlations between school-based distress and anxiety [25,26,27,28,29,30,31,32,33,34,35] and between school-based distress and two different forms of anxiety: school anxiety [42,43] and test anxiety [10,36,37,38,39,40,41]. It should be noted that this research revealed a small magnitude for all correlations, except for the association of AA, SA, and GA with the emotional manifestation of school-based distress, which showed a moderate magnitude. This manifestation of distress refers to feelings such as frustration, upset or anger. In this sense, a previous study recruited a Spanish sample of children in the 3rd to 6th grades of primary education, and found a positive and significant correlation of AA, SA, and GA with anger [52]. Therefore, the present work supports the evidence that both generalized anxiety and anxiety to situations in the school setting are related to emotional discomfort in late childhood [59]. 

Secondly, this study tried to complement the results from differential relationships between both variables with results from a person-centered approach in order to consider students’ reality in a greater extent [60]. Thus, it was examined whether there were different anxiety profiles that showed different levels of sources and manifestations of school-based distress. Hypothesis 2 is supported by the three-profile model from LPA, which included two profiles with the same weight obtained by AA, SA, and GA (Low Anxiety and High Anxiety) and a specific profile with different weight showed only by SA (High School-based performance Anxiety). This model coincides with that obtained by Fernández-Sogorb et al. [52] but differs from the four-profile model obtained by Fernández-Sogorb et al. [50] and Fernández-Sogorb et al. [51]: Low Anxiety, Moderate Anxiety, High Anxiety, and High Anxiety School-type (i.e., AA and SA). Given that all these works administered the VAA-R to samples of Spanish students enrolled in the four last grades of primary education (i.e., 3rd to 6th), the differences found among the profile solutions could be explained by the technique used. In both the research by Fernández-Sogorb et al. [52] and the present study, LPA was performed, whereas *K*-means and Latent Class Analysis (LCA) were executed by Fernández-Sogorb et al. [50] and Fernández-Sogorb et al. [51], respectively. LPA constitutes a variant of LCA that is considered more appropriate than traditional techniques, since classifies each subject into the most suitable latent profile according to their responses for categorical and continuous variables or for continuous variables [61,62,63]. In addition, we used LPA instead of LCA in this study, since “in LCA […] the latent classes are described by the differing posterior probabilities (i.e., specified after the class solution has been extracted) of endorsing each indicator variable based on class membership. In contrast, […] the resultant best fitting LPA solution is described by the different mean scores on each indicator variable, depending on class membership” [64] (p. 148). In terms of interpretability, the three-profile model from LPA showed high scores in the factor referred to anxiety-provoking social situations (i.e., SA). This specific profile (i.e., High School-based performance Anxiety) is in consonance with the scientific literature highlighting that social anxiety symptoms are characteristic of late childhood, when peer interactions gain importance (e.g., [65]. Therefore, this research has allowed a better understanding of the specificity of anxiety profiles, since it supports the tendency of Spanish students in late childhood to show high or low levels in all forms of anxiety examined or to manifest high levels of anxiety in social situations. Nevertheless, the study of profiles of child anxiety at the international level remains poorly understood, in the sense that only one work obtained a specific profile called Academic Anxiety by analyzing other forms of anxiety in British population between 11 to 13 years [48].

With regard to the differences among the profiles of child anxiety in the sources and manifestations of distress, the results support Hypothesis 3 since children grouped into High Anxiety scored significantly higher in TI, AS, ASC, and PH (with moderate effect sizes) than those grouped into Low Anxiety. The High Anxiety profile also scored significantly higher in PI, E, and B but, in this case, with large effect sizes. This finding is consistent with the positive and significant correlations found between anxiety and school-based distress in previous literature (e.g., [35,36,43]) and in this study, evidencing that students with high levels of anxiety tend to be more sensitive to stressors in the school setting and to manifest distress symptoms. Likewise, the present work provides interesting information from the person-centered approach used. Thus, no statistically significant differences were identified between children grouped into High Anxiety and those grouped into High School-based performance Anxiety in TI, AS, ASC, and PH. The students classified into the High Anxiety profile showed significantly higher scores only in PI and B (with small effect sizes) and E (with a moderate effect size). This implies that children who feel high levels of anxiety about performing in social situations tend to manifest less feelings of frustration, upset or anger, less behaviors toward others as fighting, and less negative perceptions of their classmates than children with high AA, SA, and GA. It is important to note that the research of anxiety profiles from the VAA-R by Fernández-Sogorb et al. [52] obtained similar results, even when the authors examined the possible differences among the three profiles in another construct: aggression. The students grouped into High School-based performance Anxiety showed anger, negative thoughts about others, and physical and verbal aggression to a lesser extent than children classified into High Anxiety. It can be explained by considering the autonomic nervous system (ANS), which “produces sympathetic arousal that activates most of our bodily functions in a stress situation, such as the heart and lungs” [66] (p. 17). Thus, when people experience distress, their ANS is altered, which leads to sympathetic activation [67]. The sympathetic nervous system (SNS) “responds to threat with ‘fight or flight’ action tendencies” [68] (p. 1136), with anger and anxiety being common emotional responses. Therefore, children tend to experience negative stress and anxiety by activating the same bodily functions. However, students with school-based distress can respond to stressors with “fight action tendencies”, that is, with anger.

To summarize, approximately 40% of the Spanish sample was grouped into the highest anxiety profile configured by the LPA. Students of this profile (i.e., High Anxiety) manifest negative thoughts about other students, emotional discomfort, and misbehavior to a greater extent than those who were classified into High School-based performance Anxiety (approximately 10% of the Spanish sample), probably because of a tendency to judge and react impulsively [69]. These profiles do not differ significantly from each other for any of the remaining manifestations and sources of school-based distress (i.e., physiological manifestations of distress, negative perceptions of teachers, academic stress, and poor academic self-concept). Although students with high levels of anxiety in social situations seem to have more adaptive thoughts about peers and emotional and behavioral responses, attention must be paid to these children. They could be characterized by self-focused attention, which consists of a process of heightened observation on one’s thoughts, emotions and sensations when social interactions are occurring [70], and by negative self-perceptions of performance in such social situations [71,72]. Therefore, this research has identified two risk profiles represented by approximately a half of the child population analyzed. These children need specialized intervention in order to foster their mental, emotional, and physical health [21].

Certain limitations of this work should be addressed. In the first place, this research has contributed to clarify the specificity of profiles of child anxiety at national level, but the results obtained cannot be compared with international findings since these are scarce and the forms of anxiety examined were different. Therefore, the analysis of anxiety profiles by using the VAA-R in other countries would provide relevant information for understanding the development of AA, SA, and GA in late childhood according to the geographical origin. Furthermore, the lack of consensus at international level on the specificity of child anxiety profiles also requires additional study of relevant phenomena such as sympathetic activation, in which anger is also involved. In the second place, our results do not allow to know the possible causes of high levels of anxiety. For instance, previous research suggests that parental occupation (e.g., belonging to working mothers) and parental education make influence on children’s levels of anxiety (e.g., [22]). Therefore, external factors such as family should be examined in order to get a better understanding of the development of risk profiles of anxiety. In addition, considering that anxious symptoms arise during preschool time and that scientific literature on the developmental course of anxiety from this educational stage is limited [73], future works should extend the examination of profiles of these three forms of anxiety (i.e., AA, SA, and GA) to preschool and early grade school years. In the third place, this research is pioneer on assessing the relationship of forms of anxiety with sources and manifestations of distress by using a person-centered approach, and a community sample was used for that purpose. Therefore, future studies should be conducted with clinical samples in order to verify whether the results obtained in this work can be replicated. Last, the design used does not allow establishing causal relationships between the forms of anxiety and the sources and manifestations of school-based distress examined. It could be solved by means of longitudinal data or structural equations.

Despite the mentioned limitations, our study findings are consistent with the general literature in this area: many school children experience emotional, mental, and behavioral problems that interfere with their success and happiness and prepare them poorly for adulthood. Thus, the present study provides several practical implications. On the one hand, given that around the 50% of the Spanish children examined are in risk because of their anxiety profile and their poor ability to cope with challenges in the school setting, the educational community and the psychologists specialized in this field are encouraged to use a mindfulness-based stress reduction (MBSR) program. Specifically, mindfulness meditation should be practiced each week by groups and, at the same time, at home regularly. In this sense, mindful parenting training would allow parents to collaborate by being more comprehensible in their parenting and, consequently, contributing to reducing their children’s psychopathology [74]. Teachers also need specific training in how to organize and guide mindfulness sessions. Thus, MBSR is “associated with increasing interest and availability of mindfulness teachers training courses” [75] (p. 1687). It is important to note that teachers are encouraged to deliver MBSR sessions “in a way that suits their class and timetable” [76] (p. 20) for not becoming overwhelmed by too many tasks. It should be noted that MBSR was effective in reducing students’ academic stress and emotional symptoms such as anxiety, anger or frustration, and improving children’s self-control, peer interactions and academic self-concept [77,78]. These works highlight, as a potential of this program, that it trains students to observe their thoughts without judgement, to accept stressors or anxiety-provoking situations, and to self-regulate their reactions. Considering that both risk profiles are characterized by negative perceptions of one’s academic performance, which lead to poor academic self-concept, and that children grouped into High School-based performance Anxiety tend to perceive their performance in social situations as deficient, educational professionals should integrate positive psychology into MBSR sessions. Thus, the children would learn to focus on positive aspects of their performance, their state of eustress being enhanced [79].

In addition, several innovative interventions have appeared in the treatment literature which offer hope for prevention and treatment that are safe, efficient, effective and—of special interest—can be taught for self-use by the students themselves. These tools will be described in the article in ways that allow them to be incorporated into the school curriculum, studied with simple measurements, and compared to other interventions. Because of their user-friendly and simple-to-learn formats, they can be taught to virtually all students without preliminary assessment or evaluation of a child’s particular needs. When practiced as daily habits, the tools become resources for preventing future emotional and behavioral problems. In the first place, emotional freedom techniques (EFT) have proven to be highly effective for a wide variety of samples, including school-aged children [80]. Each student “engages in the somatic tapping process on acupoints on the body while they repeat a shortened phrase to stay engaged (e.g., feel angry). This is called the ‘Reminder Phrase.’ The tapping sequence uses 8 acupoints on the face and upper body” [81] (p. 2). An advantage of this tool is that children can self-treat once they learn the procedure. In the second place, HeartMath breathing techniques also enhance psychophysiological self-regulation. Several studies have demonstrated that this training has a positive effect on children’s academic achievement and emotional wellbeing by reducing students’ levels of anxiety (see Harney [82] for a review). Last, given that anxious and angry children are likely to be traumatized, eye movement desensitization and reprocessing (EMDR) therapy is recommended. “EMDR combines imagined exposure, cognitive and psychoanalytic techniques. The main feature of the EMDR is the bilateral stimulation while the client imagines the worst part of the trauma” [83] (p. 77). The results from recent systematic reviews and meta-analyses support EMDR as an effective therapy for children with posttraumatic stress disorder and trauma-related symptoms such as anxiety [84,85,86]. 

On the other hand, Attention Training (AT) could be used with children who show High School-based performance Anxiety, since its efficacy to develop flexibility of attentional focus by reducing self-focused attention and enhancing attentional deployment has been widely demonstrated in subjects with social anxiety symptoms [87,88,89]. Finally, the results of this research support the suggestions of Gonzálvez et al. [90] to Spanish educational professionals: “to introduce a favorable working atmosphere in their day-to-day practice […] and to improve the students’ level of aspiration by promoting attributions to effort exerted and by maintaining appropriate expectations based on their level” (p. 374). In this line, the development of a positive classroom social climate and students’ self-confidence must be a priority to reduce symptoms of anxiety and school-based distress.

## 5. Conclusions

Two risk profiles of anxiety in late childhood have been identified in Spanish population. In the first place, the highest anxiety group manifests higher levels in all sources and manifestations of distress than the lowest anxiety group, which is in consonance with the positive and significant correlations identified between anxiety and school-based distress. In the second place, students characterized by high anxiety about performing in social situations probably have self-focused attention and negative perceptions of one’s own social performance, since they show fewer negative perceptions of other children, less emotional discomfort, and less misbehavior than the highest anxiety group. The results suggest that these risk profiles should be considered in order to apply specific programs that enhance children’s health.

## Figures and Tables

**Table 1:** Sample distribution by gender and age.

Gender	Age				Total
	8	9	10	11	
Boys (%)	74 (9.8%)	97 (12.8%)	87 (11.5%)	118 (15.6%)	376 (49.7%)
Girls (%)	82 (10.8%)	114 (15.1%)	79 (10.5%)	105 (13.9%)	380 (50.3%)
Total	156 (20.6%)	211 (27.9%)	166 (22%)	223 (29.5%)	756 (100%)

**Table 2:** Correlations between the forms of anxiety of the Visual Analogue Scale for Anxiety—Revised (VAA-R) and the sources and manifestations of distress of the School Situation Survey (SSS).

	AA	SA	GA
TI	0.25 **	0.22 **	0.17 **
AS	0.16 **	0.21 **	0.18 **
PI	0.27 **	0.22 **	0.19 **
ASC	0.23 **	0.22 **	0.11 **
E	0.40 **	0.31 **	0.36 **
B	0.27 **	0.22 **	0.19 **
PH	0.18 **	0.18 **	0.24 **

** *p* < 0.001; AA = anticipatory anxiety; SA = school-based performance anxiety; GA = generalized anxiety; TI = teacher interactions; AS = academic stress; PI = peer interactions; ASC = academic self-concept; E = emotional; B = behavioral; PH = physiological.

**Table 3:** Fit indices for the different profile solutions of the Latent Profile Analysis (LPA).

Model	AIC	BIC	Entropy	LRT	BLRT	Size
2 profiles	6130.62	6176.90	0.70	0.00	0.00	0
3 profiles	6002.72	6067.52	0.76	0.03	0.00	0
4 profiles	5947.80	5947.80	0.81	0.13	0.00	1
5 profiles	5904.99	6006.80	0.85	0.06	0.00	1
6 profiles	5894.79	6015.12	0.78	0.67	0.00	1

AIC = Akaike Information Criteria; BIC = Bayesian Information Criteria; LRT = Vuong-Lo-Mendell-Rubin Likelihood-Ratio Test; BLRT = Bootstrap Likelihood Ratio Test.

**Table 4:** Means, standard deviations, and post hoc contrasts between mean school-based distress scores obtained by the profiles of child anxiety.

Variable	Low Anxiety		High School-Based Performance Anxiety		High Anxiety		Statistical Significance and Effect Sizes		
	*M*	*SD*	*M*	*SD*	*M*	*SD*	*F*_(2753)_	*p*	*η* ^2^
TI	7.12	3.80	8.87	4.66	10.01	4.42	23.49	<0.001	*0.06*
AS	6.76	3.23	7.91	2.80	8.49	2.87	17.84	<0.001	*0.05*
PI	11.86	3.71	13.55	3.88	15.10	4.65	30.54	<0.001	*0.08*
ASC	12.16	2.93	13.38	2.83	14.12	3.20	22.88	<0.001	*0.06*
E	4.23	4.08	6.92	4.28	9.30	5.39	61.53	<0.001	*0.10*
B	3.23	3.51	4.77	3.83	6.24	4.30	28.07	<0.001	*0.07*
PH	3.05	2.57	3.85	2.64	4.48	2.77	13.71	<0.001	*0.04*

TI = teacher interactions; AS = academic stress; PI = peer interactions; ASC = academic self-concept; E = emotional; B = behavioral; PH = physiological.

**Table 5:** Cohen’s d indices for post hoc contrasts between the mean scores obtained by the profiles of child anxiety in the manifestations and the sources of school-based distress.

Variable	Low Anxiety vs. High School-Based Performance Anxiety	Low Anxiety vs. High Anxiety	High School-Based Performance Anxiety vs. High Anxiety
TI	0.42	0.74	-
AS	0.38	0.55	-
PI	0.45	0.83	0.38
ASC	0.42	0.66	-
E	0.65	1.17	0.52
B	0.42	0.82	0.37
PH	0.31	0.55	-

TI = teacher interactions; AS = academic stress; PI = peer interactions; ASC = academic self-concept; E = emotional; B = behavioral; PH = physiological.

## Data Availability

The data presented in this study are available on request from the corresponding author.
